# Importance of Correct Diagnosis

**Published:** 1901-04-15

**Authors:** H. H. Harrison

**Affiliations:** Wheeling, W. Va.


### IMPORTANCE OF CORRECT DIAGNOSIS.

BY H. H. HARRISON, D.D.S., WHEELING, W. VA.

Read before the Ohio State Dental Society, December, 1900.

There is no doubt of the fact that as the world grows older the human race grows wiser. And yet with all that has developed in science, and the wonderful progress made, we find blunders, errors and mistakes on every hand, proving that many of the same conditions still exist that were present, no doubt, even in prehistoric times. These conditions obtain in all classes and among all peoples, with the illiterate barbarian in his far-off home in the islands of the sea, with the best civilized people in the largest cities of the world, among the rich and among the poor, among professional men, business men and laborers. Mistakes are the birthright of man. Our profession comes in for its share, but it is our duty to modify its frequency as much as possible by continuity of thought and precise action.

The subject of physical diagnosis is one of the most important for the general medical practitioner, for without knowledge of the true pathological condition, the administration of therapeutical remedies is only guesswork; so, too, with the specialist, he must know the character of the disease before he can intelligently treat it.

Symptoms are sometimes very deceptive and the most astute diagnostician is liable to make mistakes, but errors may be reduced to pardonable frequency by close attention, persistent examinations and concentrated thought.

Let me speak of a very few cases which came under my observation, the better to illustrate my idea:

The first was a case of fungus hematodes (bleeding cancer), which was treated for three months as ulceration of the mucous membrane of the antrum. As a dentist, I was called to treat the case, but being young in the profession at that time, I called a physician as counsel. We agreed upon the diagnosis, but after the above time in treatment with the most approved remedies and no improvement, I began to suspect malignancy and mentioned my suspicions to Dr. Blank, who did not share my belief. We called in a noted specialist, who fully agreed with me, but the treatment was the best that could be made. The case terminated fatally, of course, and could not have been saved otherwise, even if the proper diagnosis had been made at first; but it humiliated me, for in looking over it now the case was plain enough and I should have avoided that mistake.

This emphasizes the importance of scrutinizing every symptom presented. We must not take things for granted, but be able to give a reason for our faith and action. In all cases of obscurity let every symptom and evidence be thoroughly weighed.

The next was a case of sympathetic neuralgia, diagnosed meningial irritation by a prominent and celebrated physician. The patient was a man thirty-five years old, and when I saw him, was emaciated and anemic after three months' treatment for disease of the brain. He had been suffering with paroxysms of pain at the base of the brain all this time, until appetite had departed, sleep was a myth, and he was a perfect wreck of his former self. I was called to examine an aching wisdom tooth, and upon percussion it produced this pain in the head. I repeated it several times, which satisfied me as to the cause of his brain trouble. The attending physician would not agree with me, but the tooth was removed, the pain ceased, the appetite and sleep returned and he was again a well man. This was a typical case of reflex nervous irritation, wherein the extremity of the nerve was diseased and the pain was felt at the origin of the nerve at the floor of the fourth ventricle of the brain.

This case shows the importance of physicians and dentists counseling with each other in such diseased conditions. I was fortunate to find the key to this trouble, and the physician had not considered dental influence in making up his diagnosis.

The next was a case with a bifurcated diagnosis; one physician made it a case of necrosis of the superior maxillary bone, another pronounced it cancer.

The patient, a man forty-two years old, was wearing an upper set of teeth, twelve years old. Upon the alveolar border, near the location of the cuspid tooth, were two openings in the soft tissues from which pus was escaping almost constantly, while just below the infra-orbital foramen there was a considerable enlargement, very hard and tender. I delayed my diagnosis for a few days and injected diluted tincture iodin into the openings, which soon stopped the formation of pus.

I then made a critical examination with an instrument, and obtained the desired information by the explorer coming in contact with enamel structure. Extracted a supernumerary cuspid in form of a quarter moon.

I could mention many more cases, but want to say something about other mistakes we make that are threatening the dignity of our profession even more than the above.

There has become such a craze for gold crown work that it is becoming alarming, and a check must be made soon or coming history will have great room to make a caustic criticism upon the operations made in the present era. I do not mean to say that gold crowns should not be used as a means of saving teeth, but the operators should discriminate as to when and where to place them. We all know that the highest aim in art is to approach the natural, and a gold crown placed upon a front tooth is anything but natural—it is abominable. To mar the beauty of the human face, and place the D. D. S. upon it is a stroke at the vitals of esthetic dentistry. If they should ever be used for front teeth at all it should be under the most peculiar circumstances. I can conceive of circumstances where it would be proper, but they are very rare indeed. We all make enough mistakes that are excusable, but this is not. It is a burning shame that dentists have permitted this error to become so general without sounding a public alarm.

While we are regretting the mistakes we have made, we must take some credit for the persistent efforts that are being put forth in saving teeth and roots from the cruel forceps. It shows signs of the opening bud that has been too sluggish in its development. It will be a great thing when we can get along without extracting any tooth or root. However this condition of things is much to be desired it is not with us yet, and we must make the best of it.

There are thousands of teeth sacrificed yet that could be saved. If we could all reach the point of seeing the great injury inflicted by even the loss of one tooth, at times, it would have a restraining influence for good. When we consider that mastication is the first step in the digestive process, and that imperfect digestion is at the bottom of most of the ills of life, we can get a more perfect conception of what the loss of the natural teeth means.

We make another mistake in permiting the indiscriminate use of tooth powders, pastes or mouth washes that are on sale for the public. There is just as much reason in a physician prescribing bismuth for all characters of stomach trouble, as there is for a dentist prescribing a general dentifrice. There are none made from the same formula that are universally applicable, as what suits the young may not suit the middle-aged, and does not suit the old. The changing conditions that may occur at any time in life may necessitate a change in treatment. Shotgut prescriptions are not up to the present age.

The saponaceous preparations are only appropriate where there is a strong acid reaction in the fluids of the mouth, and are injurious when patients have a gouty diathesis, or when pyorrhea is present or indicated.

This society has an honored member who has thought out and formulated a dentifrice that approaches more nearly to universal application than any I can now recall. See to it that your recommendations fill the requirements of your patients.

Whether by accident, mistake or natural cause, your patient has lost his or her natural teeth, that person should enlist your sympathy, your special interest, and you should make the strongest effort to compensate your patient for this loss. Unfortunately this is not universally the case, as is shown by the hideous monstrosities that are walking monuments in many of our streets to-day to the discredit of some dentist who has made a mistake.

I am happy to say that we have many operators who will not allow such conditions to escape from their hands, but some do, and these are the cases we should reach. This condition of things is partly due to the fact that many operators do not direct or superintend the making of the dentures that they place in the mouth, and it is only an accident if such cases fill the requirements of the patient. The prosthetic dentist can not be responsible for failure in a case that he has not seen, but the dentist who takes the impression should be, and can not afford to see his patient's face and countenance changed almost beyond recognition. Sometimes you find just such cases, even from the hands of the very best operators. This is a mistake and should not occur, for it is not excusable. Many of these hideous structures come from the advertising shops where nothing but money enters into the case, and the more the case is slighted the quicker the return and the more of it for the cost.

---

Hemophilia Causes Death.—A dental student of Brooklyn, N. Y., died from hemorrhage of the nose and gums. The first attack occurred three months ago, and have recurred at intervals since with increasing severity. It was impossible to prevent the attacks.

